# Biosynthesized Silica Nanosuspension as Thermal Fluid in Parabolic Solar Panels

**DOI:** 10.3390/e23020142

**Published:** 2021-01-25

**Authors:** Enrique Corzo-Deluquez, Lina Pineda-Muñoz, Adiela Ruíz-Chamorro, Carlos Ocampo-López, Margarita Ramírez-Carmona, Leidy Rendón-Castrillón

**Affiliations:** Centro de Estudios y de Investigación en Biotecnología (CIBIOT), Facultad de Ingeniería Química, Universidad Pontificia Bolivariana, Circular 1ª No. 70-01, Medellín 050031, Colombia; enrique.corzo@upb.edu.co (E.C.-D.); lina.pineda@upb.edu.co (L.P.-M.); adiela.ruiz@upb.edu.co (A.R.-C.); carlos.ocampo@upb.edu.co (C.O.-L.); leidy.rendon@upb.edu.co (L.R.-C.)

**Keywords:** parabolic solar panels, nanofluids, silica nanoparticles, biosynthesis, solar energy materials

## Abstract

In this work, the production of biologically synthesized silica nanoparticles was proposed to prepare a nanosuspension as a thermal fluid in parabolic solar panels at the laboratory level. Silica nanoparticles were produced from construction sand in two stages. Biosynthesis broth was produced by *Aspergillus niger* aerated fermentation in a 1 L bioreactor for 9 days. Each supernatant was contacted with 18% construction sand in a 500 L reactor with mechanical agitation, at a temperature of 25 °C, and a contact time of 30 min. Subsequently, the separation process was carried out. For day 9, a pH value of 1.71 was obtained as well as acid concentrations of 15.78 g/L for citrus and 4.16 g/L for malic. The metal extraction efficiency of Si nanoparticles was 19%. The vibration peaks in the FTIR were characteristic of the presence of silica nanoparticles in wavenumbers 1020 cm^−1^ and 1150 cm^−1^. Finally, a prototype solar radiation test bench for parabolic systems was built and provided with a radiation source that falls on a translucent pipe that transports the nanoparticles, which has a pump and a series of thermocouples. The heat capacity of the biotechnologically produced silica nanoparticle suspension was 0.72 ± 0.05 kJ/kgK, using material and energy balances in the flow circuit.

## 1. Introduction

Global energy consumption is underpinning a growing level of demand that will reach 778 Zettajoules by 2035 [1]. Factors such as industrialization, commercial development, and the massive use of electronic devices increase the dependence on energy sources by 1.52% per year [2,3], thereby affecting global environmental conditions in terms of CO_2_ [4,5,6], and other greenhouse gases [7].

Solar energy as a source of energy can cover a large portion of global consumption. Existing solar energy technologies include photovoltaic panels, thermal collectors, and hybrid photovoltaic/thermal panels [8,9]. However, photovoltaics technologies incorporate toxic metallic materials that include Germanium, Chromium, Gallium, and Cadmium [9,10].

Solar thermal collectors are a feasible alternative to photovoltaics technologies since they capture solar radiation and store it in thermal fluids and then transfer the heat to steam generating systems [11]. The recent research to optimize thermal collectors is focused mainly on the increase of heat transfer via structural design or improving the inner convection coefficient by increasing the thermal conductivity of the base fluid, incorporating chemically synthesized nanoparticles in the form of nanosuspensions, and applying sonication at the laboratory level [12]. The most studied base fluids are deionized water, oil, ethylene glycol, and polymer solutions. The most suitable nanoparticles comprise silica modified with copper [13], Cu–Ag/biochar [11], copper oxide, aluminum oxide, carbon nanotubes, or heavy metals such as nickel, iron, zinc, gold, and silver [14].

Although these solutions are effective, obtaining nanoparticles via a chemical process consumes high amounts of energy and generates residuals that can be toxic to people and ecosystems. Biological methods are an alternative to synthesize green nanoparticles [14], especially those that contain silica. Nanoparticles are synthesized by different microorganisms, including bacteria, yeasts, fungi, and viruses. Studies include minerals, solutions, or metal oxides [15]. In addition, microorganisms such as fungi are used in the bio-dissolution of valuable metals in solid matrices [16], and studies have been mainly focused on medical applications [17].

As we found in our literature review, no studies report the direct application of biosynthesized silica nanoparticles from a fermented broth in parabolic solar panels. The interaction between the biomolecules such as the amino acids, organic acids from the fermentation, and nanoparticles could favor the stability of the suspension, thereby avoiding the nanoparticle sedimentation [18].

Additionally, in cited studies, the analysis of the thermal improvement of nanofluids is performed using empirical observations, and few thermic models are applied to develop predictive mathematical models that are useful in the design and implementation of pilot parabolic solar panels. 

Based on the current situation, this paper proposes the biological synthesis of silica nanoparticles from a fungal fermentation and construction sand to generate a nanosuspension that serves directly as thermal fluid in parabolic solar panels. 

Finally, to evaluate the thermal conductivity of the nanosuspension, a prototype test bench for solar radiation was proposed, as well as a mathematical model based on differential equations for the thermal analysis.

## 2. Materials and Methods

### 2.1. Silica Nanoparticles Biosynthesis

The biosynthesis of silica nanoparticles was carried out in two stages. In the first, a fermented broth rich in extracellular proteins, enzymes, and organic acids was produced by a biological process and in the second, the fermented broth was brought into contact with sand as a source of silica for its extraction and biosynthesis of nanoparticles.

Initially, duplicate fermentation was carried out in a liquid state in a 1 L bioreactor and a working volume of 750 mL, using a 10% inoculum of *Aspergillus niger* in culture medium proposed by Papagianni [19] at 200 rpm, under constant aeration, and without adjustment of the initial pH for nine days.

The kinetics of the fermentation was performed by reading the pH and quantifying malic acid, citric acid, and sucrose using the HPLC technique with a mobile phase of sulfuric acid at 4 mM, with a flow of 0.52 mL/min and 70 °C in an oven. The column used was Monosaccharide H + Phenomenex, using 10 µL per sample injection. Detectors were employed at a 210 nm wavelength.

After the fermentation, the separation process of a fraction of the broth was carried out for day 5 and day 9 of the fermentation and each supernatant was contacted with 18% construction sand (average diameter 0.125 mm, #115 mesh, SiO_2_ concentration 96.21%) in a 500 L reactor with mechanical agitation, at a temperature of 25 ºC and contact time of 30 min. Subsequently, the separation process was carried out by centrifugation at 3500 rpm for 10 min. 

Silica nanoparticles were analyzed by SEM/EDS. A dry sample of supernatant was subjected to NeoScopeTM JCM-6000 Plus equipped with an Energy Dispersive X-ray Spectrometer (EDS) for elemental analysis at 15 kV. 

The supernatant was analyzed by FTIR spectrophotometry between 4000 to 400 cm^−1^ to determine the production of Silica nanoparticles. For this analysis, two samples were evaluated: the one on day 5 with a pH value of 2.62 and the one on day 9 with a pH value of 1.71.

The silica concentration of the process was quantified from the aqueous phase and the sand after acid digestion by UV-VIS spectrophotometry using the standard molybdate method [20].

### 2.2. Solar Panel Bench

A prototype radiation test bench was built, consisting of a wooden body in the shape of a 28 cm × 28 cm × 115 cm cubic parallelepiped, insulated on its walls, and lined with a reflective aluminum material. Inside, a parabolic mirror was designed and installed to cover the front face of the test bench with a focal length (P) of 1.8 cm, which describes a line according to Equation (1):

(1) $$X\;=\;\frac{Y^{2}}{4P}$$

where *P* is the focal length and 4*P* equals the length of the aperture, *Y* is the vertical coordinate axis, and *X* is the horizontal coordinate axis.

**Figure 1:**
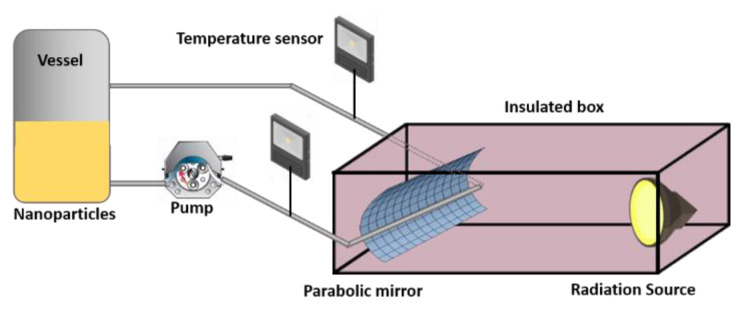
Prototype test bench for solar radiation.

A translucent pipe was installed along the focal axis that transports the nanoparticles, which are powered by a peristaltic pump. The temperature was monitored using a pair of K-type thermocouples and the flow was determined by volumetry using a test tube. The tests were performed in triplicate to assess their statistical variation. Figure 1 shows the prototype test bench for solar radiation.

The heat capacity of the silica nanoparticle suspension produced by material and energy balances in the flow circuit was determined, using the first law of thermodynamics in conjunction with a model for internal convection using Equations (2)–(4) based on the model proposed by [21]:

(2) $$\frac{dT_{f}}{dz}+AT_{f}\left(z\right)\;=\;B,$$

(3) $$A\;=\;\frac{\pi \;dr\;h_{nf}}{\dot{m}C_{p,nf}},$$

(4) $$B\;=\;\frac{\pi \;dr\;{\left[0.25I_{s}+h_{nf}Tw\right]}_{}}{\dot{m}C_{p,nf}},$$

where *d_r_* is the pipe diameter, *h_nf_* is the heat transfer coefficient W/m^2^ K, *I_s_* is the Source radiation intensity, W/m^2^, m is the the mass flow of the nanofluidic stream, kg/s, *T_w_* is the Temperature of the nanosuspension, *T_f_* is the Temperature of the fluid, *z* represent the axial direction, and *C_p,nf_* is the Heat capacity of nanofluids kJ/kgK.

## 3. Results and Discussion

### 3.1. Silica Nanoparticles Biosynthesis

In this research, we worked with *Aspergillus niger* because of its capability to synthesize a wide range of nanoparticles. The cultivation and the bioconversion can be controlled to obtain the desired nanoparticles [18]. Furthermore, *A. niger* solubilizes metals [16] due to the production of organic acids, mainly citric, gluconic, and oxalic acids as its primary metabolites during its development. These bio-produced acids are the most critical leaching agents in the process [22].

**Figure 2:**
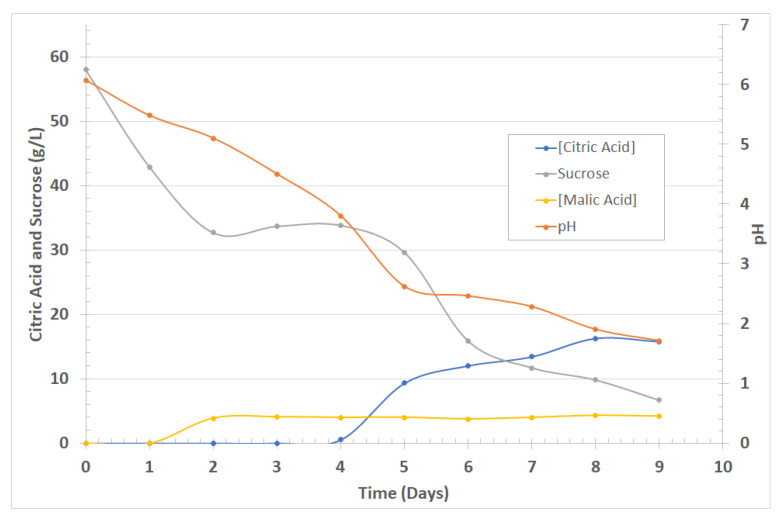
Kinetics for the fermentation process.

Figure 2 shows the kinetics of the fermentation process, showing that the *Aspergillus niger* requires a nutrient medium that stimulates its proliferation. It is observed that an increase in sucrose within the medium is directly related to greater excretion of organic acids. Similarly, the sucrose concentration decreases as it is consumed by the fungus, generating an increase in the biomass or metabolites produced [23,24].

Sucrose was used in this research because it is the most affordable carbon source for sugars such as fructose, lactose, and galactose. *A. niger* has a potent mycelial-bound extracellular invertase that is active at low pH values and hydrolyzes sucrose rapidly [25].

The production of organic acids (citric and malic acid) was the main leaching agent, resulting from the metabolism of *Aspergillus niger*. However, *A. niger* is also capable of excreting other types of acids, which leads to multiple dissolution mechanisms of mineral species [26].

Figure 2 shows the results of the fermentation. On day 5 citric and malic acid were 9.31 g/L and 3.99 g/L, respectively, and the pH was 2.62, evidencing the affinity between the substrate and the microorganism; at this stage, the production of citric acid rose uniformly. During day 9 the pH was 1.71, and acid concentrations remained constant, indicating the end of the bioprocess. The silica extracted after contacting the sand with the fermented broth was 19%, using both the fifth- and ninth-day broth. This result was verified by UV/Vis spectrophotometry using the standard molybdate method [20].

Organic acids possess interesting properties as potential leaching agents, although they cannot be applied to all minerals due to the limitations associated with their chemistry. They are presented as an alternative to consider in the future [23]. In general, organic acids are less corrosive, allow greater selectivity, and can be easily obtained from the processing of materials found in nature [26]. Finally, organic acids are presented as interesting options for leaching from different species [27].

Organic acids are weak acids, which are partially ionized in water [28]. The acidity of carboxylic acids is based on the greater polarity of the O-H bond in the center of reactivity of the molecule and the resonance stabilization of the carboxylate anion after ionization or neutralization of the acid [24,29,30,31].

These organic acids can be obtained as a product of the metabolic activity of different microorganisms, some of them capable of synthesizing several organic acids simultaneously. Species like *B. megaterium, B. Circulans*, and *A. niger* can produce these acids. However, *A. niger* is considered to be the most affordable for metal recovery [23,24].

The mechanism associated with this phenomenon could be explained when the protons resulting from the dissociation of carboxylic acid allowed the dissolution of species; then as a result of this chemical dissolution process, the formation of stable complexes could occur, where the dissociated carboxylates form stable complexes with the cations in solution, which drives greater dissociation of organic acids or an electrochemical dissolution mechanism. Thus, carboxylates react by exchanging electrons with suspension components [32].

Organic acids experience limitations in their dissociation at relatively low concentrations since the dissolution of mineral species is attributed to the presence of carboxylic ions in solution, influencing the process kinetics and the degree of extraction of metallic species [33]. However, there are other factors associated with the solubilization process of metals, such as amino acids (aspartic acid, histidine, serine, alanine, and glycine) [34] and enzyme activity that is directly correlated to the production of organic acids [35].

The fungi additionally perform the biosynthesis of nanoparticles due to the presence of bioactive metabolites, high accumulation, and improved production. Fungi react differently with metal ions for the synthesis of metal nanoparticles. The synthesis method in this work is extracellular, where amicroorganism secretes the enzyme reductase used in the bioreduction of metal ions into metal nanoparticles [18].

In general, microorganisms use various mechanisms for the synthesis of nanoparticles, including the formation of metal complexes, extracellular precipitation, changes in solubility, biosorption, toxicity through oxidation-reduction, the absence of specific transporters, and efflux pumps [18].

**Figure 3:**
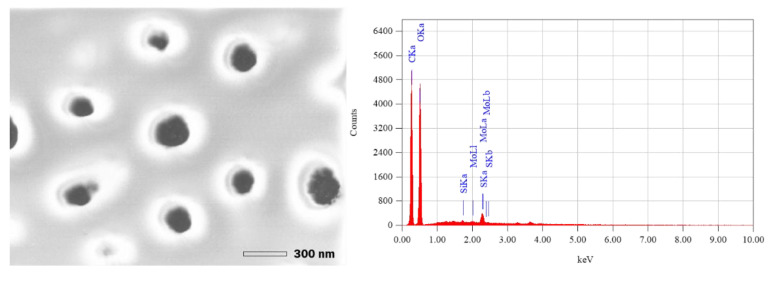
SEM/EDS analysis for nanoparticle suspension obtained with sand in contact with the fermented broth from *A. niger* at a pH of 1.71.

Scanning electron microscopy images of the dried silica nanoparticles showed mostly spherical particles of a size below 300 nm, as shown in Figure 3. 

Elemental mapping of nanoparticle samples shows the presence of Silicon at 1.739 keV (0.15 ± 0.01% mass). Other compounds such as Carbon (40.21 ± 0.10% mass), Oxygen (57.34 ± 0.26% mass), and Sulfur (0.48 ± 0.02% mass) are present due to the interaction of the construction sand and biomolecules from the fermented broth to produce the nanoparticles.

**Figure 4:**
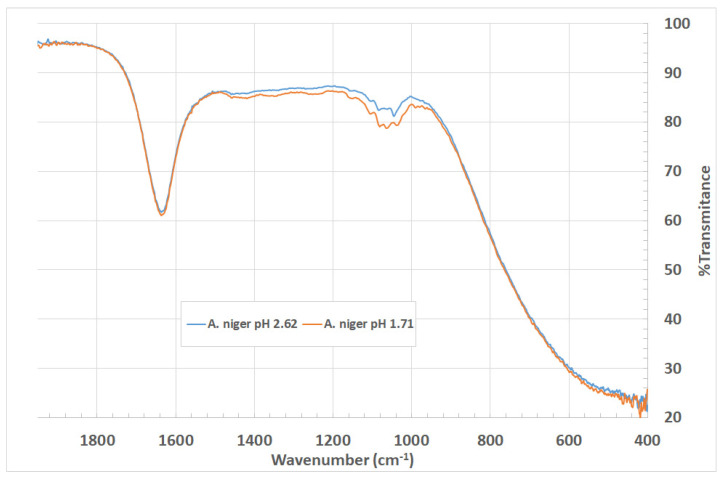
FTIR spectrum for nanoparticle suspension obtained with sand in contact with the fermented broth from *A. niger*. Samples were obtained at pH values of 1.71 and 2.62.

Figure 4 shows the FTIR spectra of the liquids coming from the treatment between the sand and the fermented broth from *A. niger*. It presents two samples at different pH values: 1.71 and 2.62.

Fourier transform infrared spectroscopy (FTIR) analysis is used to characterize and confirm the presence of nanoparticles [18]. The spectra shown in Figure 4 are similar to the results obtained by Li et al. in the production of Si nanocomposites in resin matrices [36].

As shown in Figure 4, there are peak characteristics of silica nanoparticles. In the fingerprint region, the wavenumbers 1020 cm^−1^ and 1150 cm^−1^ represent the asymmetric stretching vibrations Si-O-Si. The weak bands at 900 cm^−1^ and 949 cm^−1^ are possibly attributed to the symmetric stretching vibrations Si-OH and Si-O-Si in amorphous silica. Among the two treatments, a pH of 1.71 exhibits higher absorbance, which reflects a higher concentration of silica nanoparticles in quantitative terms.

The results showed that the broth produced by *Aspergillus niger* at pH 1.71 provides the best conditions to produce Silica nanoparticles, thereby coinciding with the conditions reported by Ovais et al. on the importance of pH in the production of citric acids from the fermentation of *Aspergillus niger*, and its contribution in the formation, size, and structure of nanoparticles [18].

The production of Silicon (Si) nanoparticles, using *Aspergillus niger*, is believed to occur due to the production of extracellular enzymes such as acetyl xylan esterase, cellobiohydrolase D, glucosidase, and β-glucosidase, which are relevant in the biological synthesis of metallic nanoparticles [37].

*Aspergillus niger* was used to produce ZnO nanoparticles as an antibacterial potential, to degrade inks [38], and to produce cobalt oxide nanoparticles [39].

Different filamentous fungi are satisfactory for the biosynthesis of gold nanoparticles. Fungal compounds and media composition play a role in stabilizing nanoparticles. Furthermore, the fungal proteins are responsible for the production of nanoparticles [18].

Bioreduction is a green method for the synthesis of metallic nanoparticles. Through this approach, the plant extracts or the microorganisms used for the reduction of the metal salt are converted into nano-sized, non-toxic, and stable metals [15].

Nanoparticle biosynthesis is competitive due to its easy production and scaling, as well as its well-defined morphologies, and improved biocompatibility if compared with physiochemical-based nanoparticles, making it more effective than nanoparticles synthesized by non-biological means [18].

### 3.2. Parabolic Solar Panels

**Figure 5:**
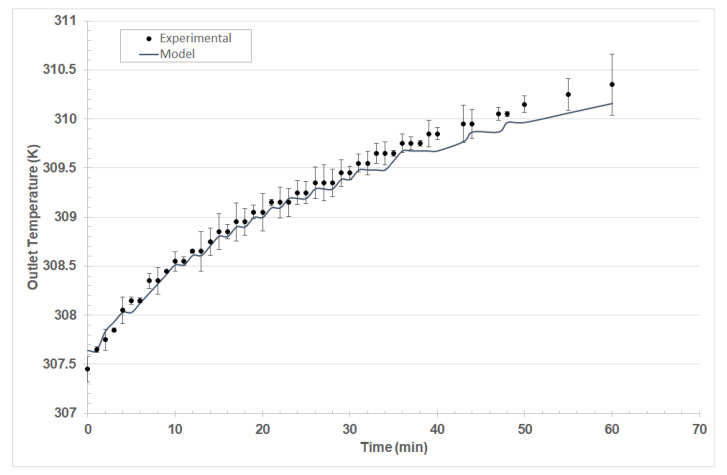
Energy balances used in conjunction with Equations (2)–(4) to predict the outlet temperature of the solar panel bench.

As shown in Figure 5, the thermodynamic model, based on Equations (2)–(4), was accurate for predicting the thermal behavior of the solar test bench. In the monitoring of the temperature for thermal fluid, a progressive increase in the outlet temperature was evidenced. When adjusting the parameters, the heat capacity was used as a correlation parameter between experimental data and the model, minimizing the total square error in the MS Excel solver.

Solving Equations (2)–(4), a heat capacity of 0.72 ± 0.05 kJ/kgK was obtained for the nanosuspension, which is within the expected range of commercial nanoparticles obtained by chemical synthesis between 0.714–0.765 kJ/kgK, according to the results presented by Bait [21].

On the other hand, the thermal conductivity of nanofluids increases with increasing temperature and volume fraction [13]; therefore, its application in the design of thermal solar panels is highly relevant.

Since heat transfer is a surface phenomenon between the interface of the fluid and the particles, the heat transfer rate will increase significantly on a nanometric scale by increasing the surface area of the nanoparticles, consequently providing an increase in thermal conductivity of nanofluids [13].

Several studies have shown how the nanoparticles of Al_2_O_3_, Cu_2_O, MgO, SiO_2_, TiO_2_, and ZnO are effective in increasing thermophysical properties, such as density, viscosity, the thermal conductivity, and specific heat with efficiencies of 79.9% [21].

## 4. Conclusions

The fermented broth for day 9 was revealed to be more efficient for the biosynthesis process of Si nanoparticles, presenting a pH value of 1.71 and acid concentrations of 15.78 g/L for citric and 4.16 g/L for malic acid, and peaks of vibration in the FTIR characteristic of the presence of silica nanoparticles in the fingerprint region, specifically in the wavenumbers 1020 cm^−1^ and 1150 cm^−1^ which represent the vibrations of asymmetric stretching Si-O-Si.

Biosynthesized Si nanoparticles are amorphous due to the weak bands found in the FTIR at 900 cm^−1^ and 949 cm^−1^, which can possibly be attributed to symmetric stretching vibrations Si-OH and Si-O-Si.

The SEM/EDS analysis determined that the biosynthesized Si nanoparticles are in the nanometric domain, showing spherical particles below a 300 nm diameter. 

The heat capacity of the biotechnologically produced silica nanoparticle suspension was 0.72 ± 0.05 kJ/kgK, which was obtained using material and energy balances in the flow circuit.

## Data Availability

The data presented in this study is available in the article.
